# Homegarden diversity and food security in southern Mexico

**DOI:** 10.1007/s12571-021-01148-w

**Published:** 2021-02-13

**Authors:** Jennifer Castañeda-Navarrete

**Affiliations:** Policy Links Unit, IfM ECS, University of Cambridge, 17 Charles Babbage Road, Cambridge, CB3 0FS UK

**Keywords:** Home gardens, Agrobiodiversity, Food security, Urbanisation, Mexico

## Abstract

Home gardens are recognised in the literature for their contribution to food security, yet the process by which agrobiodiversity and household characteristics mediate this relationship is less well understood. This paper contributes to fill this research gap by drawing on a multi-site case study in the Yucatán region in Mexico. By applying regression analysis, the significance of the association between home garden diversity and food security is confirmed. Plant diversity is found to have a positive association with food consumption scores and the frequency of vegetable intakes. The number of animals used for food purposes is also found to have positive and significant associations with food consumption scores and frequency of meat intakes. However, the dimension and the significance of these positive associations were found to vary among communities and quantiles of the distribution of food security measures. In the households studied, younger individuals and better-educated people, males and Spanish speakers were more likely to engage in jobs in urban areas. Engagement in urban jobs was found to involve complementarities with the overall plant diversity of home gardens, but also trade-offs with the diversity of vegetables and other herbs used for food purposes and with the abundance of animals raised for food purposes.

**Supplementary Information:**

The online version contains supplementary material available at 10.1007/s12571-021-01148-w.

## Introduction

In 2018, about 2 billion people struggled to gain regular access to sufficient nutritious and sufficient food. Among these, more than 820 million experienced hunger (FAO, UNICEF, WFP and WHO 2019). After decades of improvements in the global food security and nutrition of the world population, the last three editions of the report on *The State of Food Security and Nutrition in the World* have revealed that this trend is reversing. The most recent edition in particular highlights the rise of hunger in almost all African sub-regions, in Latin America and the Caribbean, and in Western Asia (FAO, UNICEF, WFP and WHO 2019).

Governments and development practitioners have used agricultural interventions to improve people’s food security and nutrition since the 1960s (Masset et al. 2012). Early interventions focused on increasing agricultural production and productivity, whereas more recent approaches have centred on the quality of food through fortification and production diversification (Masset et al. 2012; World Bank 2007; Ruel et al. 2018). Home gardens are an example of the latter type of interventions.

In the past two decades, home gardens have become a popular intervention among different development actors to promote increased household production of fruits and vegetables (Marsh 1998; World Bank 2007; Masset et al. 2012). Home gardens are complex agroforestry systems that exhibit diverse layers of vegetation strata, from herbs and crops, to shrubs and high trees, where domestic and wild animal components are usually integrated. In addition to their diversity, one of the most distinctive characteristics of home gardens is their proximity to the dwelling space (Fernandes and Nair 1986; Kumar and Nair, 2004).

Despite the fact that, in policy circles, the study of home gardens is a relatively recent phenomenon, these agroforestry systems have constituted a key component of rural food systems for centuries. In Southeast Asia, their origin is traced back to around 13,000 and 9000 B.C., beginning with the accidental propagation of seeds (Soemarwoto 1987). Kumar and Nair (2004) cite illustrations of home gardens in the Indian epics Ramayana and Mahabharata, which date back to 7000 B.C and 4000 B.C., respectively. Meanwhile in Mesoamerica, there is evidence of the existence of home gardens since the year 6000 B.C. (Mariaca Méndez 2012).

**Fig. 1:**
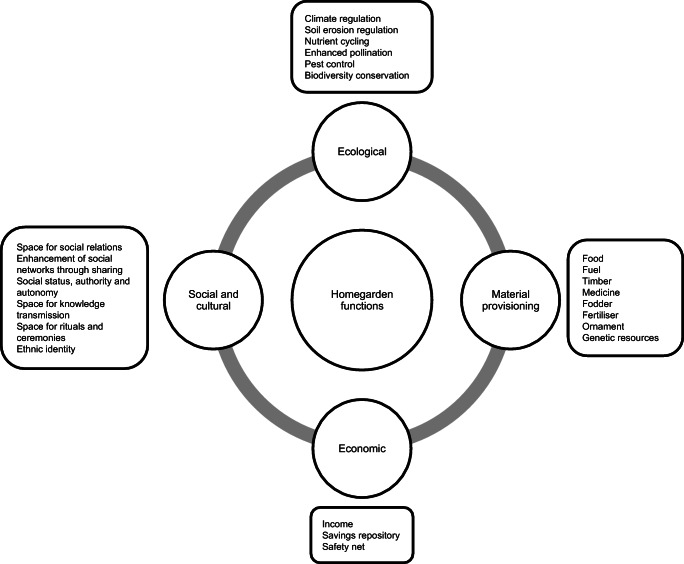
Classification of home garden functions

The benefits that households may obtain from their engagement in homegardening depend on the levels and types of species diversity that determine the different functions performed by these agroforestry systems. As Fig. 1 depicts, these include: ecological functions, material provisioning functions, economic functions, and social and cultural functions. Among these multiple functions, this research focuses on the provision of food (material provisioning), that is, on the contribution of home gardens to food security.

There is significant evidence that links home gardens to improvements in food consumption and dietary diversity. Examples include studies across several sub-Saharan countries. In South Africa, participation in home garden programmes was found to contribute to reductions in food insecurity in rural households by up to 41.5% (Tesfamariam et al. 2018). In rural Kenya, positive associations were found between the number of livestock kept in home gardens and household food security measured through food stocks and the number of meals consumed daily (Musotsi et al. 2008). Last, in Zambia, positive associations were found between production diversity and children dietary diversity and nutrition status (Kumar et al., 2015).

Trends similar to those in sub-Saharan Africa are present throughout much of southern Asia. In Bangladesh, a project promoting low-cost vegetable gardens combined with nutrition education increased household consumption of vegetables (Marsh 1998). A later study on a similar programme in this country also identified positive effects on the production of leafy vegetables and diversity of vegetable consumption (Schreinemachers et al. 2015). In rural Nepal, household production diversity, measured through the number of food groups produced, was positively associated with maternal dietary diversity, children’s dietary diversity (aged 6–59 months) and children’s weight-for-height *z*-scores (Malapit et al. 2015). Additional evidence was found in the Philippines, where children from households with home gardens were found to have higher dietary diversity scores (Cabalda et al. 2011).

The patterns described above have also emerged in several studies in Latin America. In rural Guatemala, crop and animal species richness, from milpas, home gardens and coffee plantations, was found to be associated with higher dietary diversity (Luna-González and Sørensen 2018). In rural Mexico, positive associations were found between crop diversity and children dietary diversity (aged 24–58 months) (Dewey 1981).

Positive impacts on micronutrient intakes have also been identified in evaluations of home garden programmes. Examples of these studies include Bangladesh, where long-term impacts on the micronutrient supply for iron, zinc, folate and vitamin A were identified from an integrated home garden intervention that combined training in gardening practices with nutrition education (Baliki et al. 2019). Burkina Faso, where positive impacts on mothers’ dietary diversity, as well as on intakes of fruit and meat were found from an evaluation of an intervention involving the delivery of agriculture inputs, training and educational activities on agricultural practices, health, nutrition, and hygiene (Olney et al. 2016). Indonesia, where Javanese home gardens were found to provide 18% of the calories and 14% of the protein consumed by the households studied (Soemarwoto et al. 1985). Last, Mexico, where home gardens were found to provide significant contributions to intake of nutrients, such as 10% of protein, 55% of vitamin A and 73% of vitamin C (Stuart 1993).

Despite this growing evidence, there is little information in the literature on how household characteristics and agrobiodiversity mediate the impact of home gardens on food security (Masset et al. 2012; Sibhatu and Qaim, 2018; Ruel et al. 2018). Most of the studies that have found positive results also conclude that the relationship between planned agrobiodiversity and food security is complex and dependent on household and context characteristics (c.f. Jones et al. 2014; Luna-González and Sørensen 2018; Sibhatu and Qaim 2018; Zanello et al. 2019). Additionally, a significant number of studies have found null or negative associations. From a review of 45 studies, Sibhatu and Qaim (2018) find that over 20% reported non-significant associations, while over 60% reported non-significant or negative results for sub-samples of the observations or after controlling the analysis for household and community characteristics. Sibhatu et al. (2015) also suggest that contributions of farm diversity to food security may diminish or even become negative when production diversity is already high. This pattern is explained by increasing income trade-offs as farm diversification reaches beyond optimal levels.

Studies that examine the relationship between agrobiodiversity and food security have highlighted access to markets – measured through physical distance, income or wealth, food prices, market crop diversity and transport costs – as a factor that complements production diversity and, in some cases, has an even larger effect on food security (c.f. Dewey 1981; Bhagowalia et al. 2012; Jones et al. 2014; Bellon et al. 2016; Hirvonen and Hoddinott 2017; Zanello et al. 2019).

Other variables that have been identified as significant confounding factors in determining food security outcomes include the following:

- Context characteristics such as infrastructure and remoteness (Adjimoti and Kwadzo 2018); agroclimatic conditions (Hirvonen and Hoddinott 2017); and seasonality (Zanello et al. 2019; Chávez Zander 2014; Bellon et al. 2016).
- Household characteristics such as age (Jones et al. 2014; Chávez Zander 2014; Luna-González and Sørensen 2018), education (Jones et al. 2014; Kumar et al. 2015; Luna-González and Sørensen 2018), household size (Jones et al. 2014; Kumar et al. 2015; Luna-González and Sørensen 2018), gender of the household head (Jones et al. 2014), women’s empowerment (Malapit et al. 2015), access to sanitation and cooking facilities (Dewey 1981; Kumar et al. 2015; Luna-González and Sørensen 2018), and storage facilities (Adjimoti and Kwadzo 2018).

Against this backdrop, this paper contributes to filling the knowledge gap on how species diversity and household characteristics mediate the impact of home gardening on food security. The paper draws on a multi-site case study in the southeast of Mexico and regression analysis.

## Research context and methods

### Research sites

The research was conducted in four sites located in Yucatán, a state in the southeast of Mexico. Yucatán has a population of about 1.9 million inhabitants, 16% of whom live in rural areas (Instituto Nacional de Estadística y Geografía 2010). The region is notable for its rich cultural and biological diversity. Yucatán has the largest proportion of indigenous people in Mexico who represent more than half of the Yucatecan population (Consejo Nacional de Evaluación de la Política de Desarrollo Social 2014). The main indigenous group in Yucatan are the Mayas.

Mexico is considered the 12th most megadiverse country in the world (Becerril et al. 2014). As in the rest of the country, the biological diversity of the Yucatecan region has interacted and co-evolved with its cultural richness over thousands of years as its inhabitants have transformed entire landscapes and domesticated a wide array of plant and animal species (Moreno-Calles et al. 2014). Agroforestry systems, such as home gardens, are emblematic examples of biocultural management and conservation (Moreno-Calles et al. 2014; Moreno-Calles et al. 2016; Pietersen et al. 2018).

However, similar to many other developing regions, Yucatán faces the paradox of having substantial biological and cultural richness as well as a population suffering from high levels of deprivation and malnutrition (Pingali 2007; Becerril et al. 2014). According to official statistics, 48.9% of the Yucatecan population lives in poverty, and 27% of the households face moderate to severe food insecurity (Instituto Nacional de Salud Pública 2013; Consejo Nacional de Evaluación de la Política de Desarrollo Social 2012). In addition to its children undernutrition rate, which is higher than the national average, Yucatán is the Mexican state with the largest proportion of adults between 20 and 59 years of age who are overweight or obese (over 70% of the total population of the age of reference) (Instituto Nacional de Salud Pública 2013).

Research sites were selected within the Yucatecan region to represent two historical regions: the sisal and the *milpa* regions. These two regions represent different modes of engagement in agriculture and distinct urbanisation transitions. The sisal region takes its name from the former commercial production and processing of sisal (*Agave sisalana* Perriné*, henequén* in Spanish and *ki* in Mayan), a type of agave used for textile purposes, an industry that saw its boom during the first decades of the twentieth century. This is the more urbanised region, with its heart located in Mérida, the capital city of Yucatán. In contrast, the *milpa,* or maize-growing region, has been more isolated, and its residents’ livelihoods are still highly attached to traditional agriculture. Within each region, the research sites studied represent different levels of urbanisation: Hocabá (peri-urban, sisal region), Sahcabá (semi-rural, sisal region), Yaxcabá (semi-rural, *milpa* region) and Kancabdzonot (rural, *milpa* region).

Traditionally, farming families in Yucatán, Mexico, based their subsistence on two main agroecosystems: the *milpa* (a swidden agriculture system based on maize, beans and squash as the main crops) and the home garden, complemented by forest management and apiculture (Terán and Rasmussen 1994; Jiménez-Osornio et al. 2003). However, an intensified connection with urban centres has resulted increasingly diversified livelihoods (Kay 2015).

Home gardens are considered mainly female spaces, although children and elderly people are also involved in their management and young and middle-aged men usually help with some of the heaviest tasks (Jiménez-Osornio et al. 2003; Chi Quej 2009; Dietrich 2011). The home garden is usually regarded as part of the *milpa* system, even though they are located in different plots. The *milpa* provides the main staples, while the home garden complements the diet, providing spices, vegetables, fruits and animal protein (Terán and Rasmussen 1994; Cuanalo de la Cerda et al. 1998). However, because households are abandoning the *milpa* as their main livelihood due to urbanisation transformations, home gardens are now interacting with off-farm occupations to provide means of living to the Yucatecan households (Leatherman and Goodman 2005).

### Data collection and analysis

The research in this paper is based on primary data collected over the period from September 2016 to April 2017. It draws mainly on data of a survey conducted in 316 households; however, some insights from focus group discussions and in-depth interviews are also discussed. The household survey covered the following themes: home garden characteristics, including a list of all the plant and animal species; housing characteristics; respondents’ perceptions on home garden dynamics and wellbeing meanings; socioeconomic characteristics of the household members; food consumption; and support received in the household from development actors.

Households were selected following a proportionate stratified random sampling approach. This approach facilitated the selection of households that were evenly geographically distributed. Maps of each research site were obtained and divided into four sections, so that each section contained a similar number of households. The sampling variables used were diversity measurements of home gardens from previous studies, and the sample size was computed for a confidence level of 95%.

#### Diversity data

Agrobiodiversity data was collected in order to gain a general picture of the different types and levels of home garden diversity as a proxy variable of the main functions that the home gardens perform. In the context of agricultural systems, the term ‘agrobiodiversity’ is used to refer to both planned diversity, the crops and livestock that farmers manage, and associated biota, such as soil microbes, weeds and fauna (Kontoleon et al. 2009). An inventory of planned agrobiodiversity including plant species and domesticated vertebrate animals was conducted as part of the household survey. The common species name, the number of individuals of each species and how people used it were recorded with the help of research participants.

Species richness and Shannon indices are used to describe the plant diversity of the home gardens while abundance (number) of animals used for food purposes is used to assess the animal component of the home garden. The Shannon index captures the species diversity, accounting for the relative abundance of each species in relation to the overall cropping pattern (Sunwar et al. 2006). It is calculated through the following formula:

$$H=-\sum \limits_{i=1}^n{p}_i\ln {p}_i$$

where *n* represents the total number of individuals and *p*_*i*_ represents the proportional abundance of species *i*, i.e. number of individuals of species *i* divided by *n*.

#### Food security

This research adopts the 1996 World Food Summit’s definition of food security: “Food security exists when all people, at all times, have physical and economic access to sufficient safe and nutritious food that meets their dietary needs and food preferences for an active and healthy life” (FAO 2008, p. 1). This definition implies four dimensions of food security: (i) physical availability, which is determined by the level of food production, stock levels and net trade; (ii) economic and physical access to food at the household level; (iii) food utilisation, understood as “the way the body makes the most of various nutrients”; and (iv) stability of the other three dimensions over time (FAO, 2008, p. 1). Because the focus of this research is on household dynamics, only two of the four dimensions of food security are addressed: food access and food utilisation.

Food consumption scores (FCS) are used to analyse the contribution of home gardens to food security. Based on the data collected to construct the FCS, frequency in the intake of vegetables and meat and fish is also assessed (0–7 days in the previous week). The FCS was selected as main food security measure because it captures information about the usual household diet, as it summarises consumption frequency over a seven-day period. In addition, the FCS has been validated in different contexts, including Central America (Lovon and Mathiassen 2014), and against other food security measures, such as caloric intake (Wiesmann et al. 2009). It also offers the flexibility to adapt its thresholds to the specific research context (World Food Programme 2008). This latter characteristic was particularly relevant in the research sites where high frequency of sugar and fat intakes were found.

The FCS is a composite index that captures dietary diversity, food frequency, and relative nutritional importance of different food groups (World Food Programme 2008), and it is computed “using the frequency consumption of different food groups consumed by a household during the 7 days before the survey” (World Food Programme 2008, p. 8). The steps for computing the FCS are as follows:

- Group all food items into specific food groups: main staples, pulses, vegetables, fruit, meat and fish, milk, sugar, oil, and condiments.
- Sum all the consumption frequencies of food items of the same group and recode the value of each group to a maximum of 7.
- Multiply the frequency value by the weight assigned by the World Food Programme: main staples – 2, pulses – 3, vegetables – 1, fruit – 1, meat and fish – 4, milk – 4, sugar – 0.5, oil −0.5, and condiments – 0.
- Sum the weighted food group scores (World Food Programme 2008).

#### Regression analysis

Linear, quantile, Poisson and probit regression models were estimated to analyse the relationship between home garden diversity and food security, controlling for household and community characteristics. This approach was adopted to assess the sensitivity of the results to parametric assumptions and to different points of the distribution of the outcome variable. A pooled regression of the four communities was estimated (Table 4) in addition to specific regressions of the peri-urban and rural sites and a pooled regression of the semi-rural sites (Table 5).

The dependent variables entering the models are the FCS discussed in the previous section and frequency of vegetable and meat intakes. FCS are used as dependent variables in linear and quantile regressions and a binary variable indicating whether the household is food secure or not is used in probit regressions. Frequency of vegetables and meat intakes (1 to 7 days) are modelled using Poisson and count quantile regressions. A binary variable taking the value of 0 whether the frequency of intakes was below 4 days and 1 if the frequency was 4 days or over is modelled using probit regressions.

Cluster analysis was performed to define context specific thresholds that accounted for the high consumption of sugar and fat identified in the research sites. The thresholds defined from this analysis are as follows: FCS 0–51.5 poor, FCS 52–76 borderline, and FCS > 77 acceptable. Following the common practice (Wiesmann et al. 2009; Lovon and Mathiassen, 2014), the households that reported a ‘poor’ food consumption score are considered as severely food insecure in the probit regressions.

Based on the literature review presented in the introduction and on exploratory data analysis, the independent variables included in the regression analyses are: home garden diversity, age of the household head, youth dependency ratio, average education of the adults of the household, language spoken by the household head, gender of the household head, wealth, rural–urban interactions, subsidies and location.

Since endogeneity of home garden diversity could be affecting the model (omitted variable bias), a first attempt was made in applying an instrumental variable and using the size of the plot, the age of the plot, the occupation of the household head and different combinations of these variables as instrumental variables. However, exogeneity of home garden diversity was not rejected.^1^ This does not necessarily refute the endogeneity of the variable of interest, it but may only indicate that the variables used may be weak instruments. Additionally, variance inflation factors (VIF) were computed to verify if multicollinearity (high correlation among independent variables) was affecting standard errors estimates. VIF values for all the regressors were below the ‘rule of thumb’ of 10 in the different models computed with the exception of some specifications of the regressions estimated for the rural community: quantile and probit regressions where the reported VIF values for average education and age were slightly greater than 10.

## Results

### Home garden species diversity and food security

A total number of 18 animal species and 212 plant species were recorded in the 316 home gardens surveyed. Of the plant species, 115 were herbs, 29 were shrubs and 68 were trees. As explained in the introduction, species diversity contributes to the different functions that home gardens perform and the benefits that people derive from them. An increasing peri-urban – rural gradient was observed in the diversity of plants and food animals, with the exception of Sahcabá, a semi-rural community located in the sisal region. This community showed the lowest diversity of plants among the four research sites. The main differences in the type of species diversity across peri-urban – rural gradient were found in herbs used for food purposes, which include vegetables, and the abundance of animals raised for food purposes, mainly poultry and pigs (Table 1).

**Table 1:** Biodiversity indicators of the home gardens by research site, Yucatán, Mexico

Variable	Statistic	Hocabá (Peri-urban, sisal region)	Sahcabá (Semi-rural, sisal region)	Yaxcabá (Semi-rural, *milpa* region)	Kancabdzonot (Rural, *milpa* region)	Kruskal-Wallis H *p* values
Shannon diversity index (plants)	*Median*	1.923	1.628	2.040	2.237	<0.001
Number of plant species	*Median*	8.0	6.0	11.0	13.0	<0.001
Number of species of shrubs	*Median*	0.0	0.0	1.0	1.0	<0.001
Number of species of herbs	*Median*	2.0	1.0	3.0	4.0	<0.001
Number of species of trees	*Median*	5.0	5.0	7.0	8.0	<0.001
Number of herbs used for food purposes	*Median*	1.5	0.0	4.0	10.0	<0.001
Number of animal species	*Median*	2.0	2.0	2.0	3.0	<0.001
Number of animals raised for food purposes	*Median*	0.0	2.0	3.0	8.0	<0.001

Notes: Hocabá, *n* = 98; Sahcabá, *n* = 81; Yaxcabá, *n* = 84; Kancabdzonot = 53

Source: Author’s survey data (December 2016–April 2017)

**Fig. 2:**
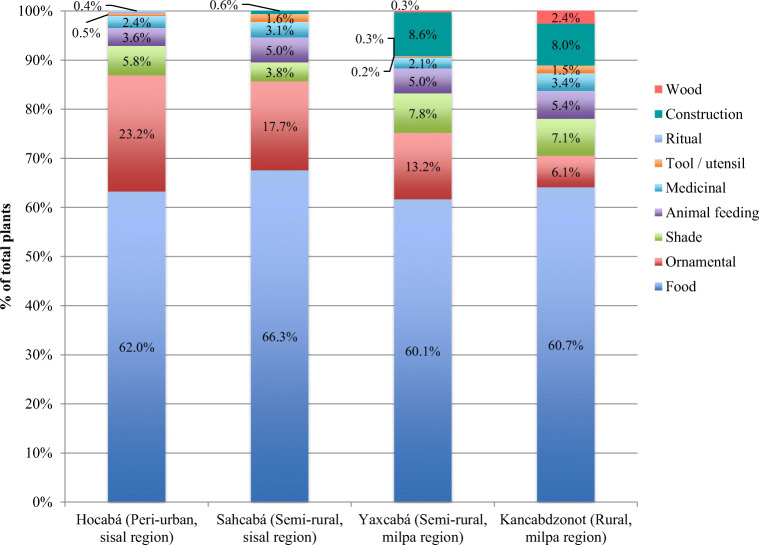
Distribution of the number of plants by use and research site, Yucatán, Mexico]

Home gardens in the research sites were mainly valued as sources of food. Over 60% of the survey respondents mentioned food consumption as the main reason for home gardening. However, home gardens also provide other materials that contribute to people’s livelihood security, including fodder, medicinal plants, timber and other construction inputs. The home gardens surveyed were identified to perform ecological, economic, social and cultural functions, and the relevance of these functions varied across the peri-urban – rural spectrum. The households located in the most rural communities had more uses for their home garden plants, including: as food, as an ornament, as shade, for feeding animals, as medicine, as tool, for rituals, in construction and as timber (Fig. 2). In addition, the ornamental role of home gardens was found to increase in importance along with urbanisation.

The main source of food in the research sites was the market, although the home garden, the *milpa,* other plots, gifts and hunting were also mentioned as primary and secondary sources of food. The dependence on the home garden as a source of food showed an increasing peri-urban – rural gradient. In the more urban communities of the sisal region, less than 30% of the households surveyed obtained at least one food group from the home garden; while in the communities of the *milpa* region, the proportion of households was above 70%.

FCS were computed to assess food security status at household level, as explained in section 2. Significant differences were observed among the research sites. Hocabá (peri-urban, sisal region) showed the highest proportion of households with borderline and acceptable food consumption, while Sahcabá (semi-rural, sisal region) showed the lowest proportion of households in these categories (*p* value<0.10) (Table 2).

**Table 2:** Proportion of households by food consumption score by research site, Yucatán, Mexico

Food consumption score	Hocabá (Peri-urban, sisal region)	Sahcabá (Semi-rural, sisal region)	Yaxcabá (Semi-rural, *milpa* region)	Kancabdzonot (Rural, *milpa* region)
0–51.5 (Poor)	9.18	24.69	13.1	22.64
52–76 (Borderline)	42.86	38.27	47.62	32.08
>77 (Acceptable)	47.96	37.04	39.29	45.28

Pearson Chi-squared: 12.004, p value: 0.062

Source: Author’s survey data (December 2016–April 2017)

Table 3 shows key characteristics of the households studied while Table 4 presents the results of the regression analysis between household food security measures (FCS and food intakes) and home garden diversity, as measured through plant species diversity and the abundance (number) of animals raised for food purposes. From the pooled regressions including the four communities the coefficient estimates suggest a positive association between plant species diversity and FCS, particularly at higher quantiles of the distribution and these quantiles overlap with the higher quantiles of the distribution of the wealth index. A positive association is also found between the number of animals raised for food purposes and FCS, but in this case in the lowest quantile, which represents the most vulnerable households. Regarding the frequency in the intake of vegetables, positive associations were found with plant diversity, but only from the probit regression. Positive associations were also identified between the abundance of animals and meat intake, from the probit regression and in the lowest quantile of the distribution.

**Table 3:** Selected characteristics of the households studied by research site, Yucatán, Mexico

Household characteristics	Statistic	Hocabá (Peri-urban, sisal region)	Sahcabá (Semi-rural, sisal region)	Yaxcabá (Semi-rural, milpa region)	Kancabdzonot (Rural, milpa region)
Land *(solar)* size (mean, squared metres)	*Mean*	1363.8	1137.1	924.1	1239.3
	*Std. Dev.*	1643.1	1138.0	783.5	970.0
Frequency in vegetables intakes (0–7 days)	*Mean*	4.7	4.3	4.6	4.1
	*Std. Dev.*	2.3	2.1	2.1	2.0
Frequency in meat and fish intakes (0–7 days)	*Mean*	5.2	4.5	5.0	4.7
	*Std. Dev.*	1.6	1.8	1.5	1.5
Age of household head (years)	*Mean*	58.8	53.1	55.0	46.7
	*Std. Dev.*	15.4	16.4	15.6	14.4
Average household education (years)	*Mean*	6.5	5.9	6.8	6.5
	*Std. Dev.*	3.6	2.9	3.5	2.6
Youth dependency ratio (Ratio of the children under 15 years old divided by the number of adults in the household)	*Mean*	0.55	0.96	0.31	0.52
	*Std. Dev.*	0.82	1.03	0.55	0.57
Female head without partner	*Proportion*	20.41	8.64	13.10	7.55
Male head without partner	*Proportion*	10.20	3.70	4.76	3.77
Household head speaks Maya	*Proportion*	0.00	2.50	4.82	13.21
Household head speaks Spanish and Maya	*Proportion*	18.37	3.75	9.64	0.00
Household head speaks Spanish	*Proportion*	81.63	93.75	85.54	86.79
Urban jobs (% of household members working in urban jobs)	*Mean*	0.30	0.35	0.16	0.07
	*Std. Dev.*	0.25	0.27	0.22	0.17
Household income, mean, adult scale equivalent MXN (GBP/USD)	*Mean*	1670.4	1545.7	1618.6	977.0
	*Std. Dev.*	1133.4	835.2	1494.4	1042.0
Wealth index (0–1, 5 assets)	*Mean*	0.54	0.44	0.44	0.45
	*Std. Dev.*	0.23	0.19	0.20	0.21
Prospera beneficiary	*Proportion*	17.35	41.25	67.86	83.02
Sixty-five and over beneficiary	*Proportion*	7.14	7.50	21.43	9.43

Source: Author’s survey data (December 2016–April 2017)

**Table 4:** Association between home garden diversity and food security (pooled regressions of the four research sites), Yucatán, Mexico

**Dependent variable: Food Consumption Score**	**OLS**		**Q (0.25)**		**Q (0.50)**		**Q (0.75)**		**Probit**	
*Shannon diversity index (plants)*										
Coefficient	3.894	**	3.250	***	5.865	***	6.104	**	0.056	**
Robust standard error	0.755		0.789		0.986		2.364		0.056	
R^2^ / Pseudo R^2^	0.138		0.119		0.115		0.067		0.162	
*Number of animals raised for food purposes*										
Coefficient	0.204		0.248	***	0.271		0.054		0.005	**
Robust standard error	0.122		0.087		0.182		0.074		0.003	
R^2^ / Pseudo R^2^	0.132		0.118		0.107		0.064		0.158	
**Dependent variable: Vegetables intake**^**1/**^	**Poisson**		**Q (0.25)**		**Q (0.50)**		**Q (0.75)**		**Probit**	
*Shannon diversity index (plants)*										
Coefficient	0.057		0.091		0.115		0.014		0.212	**
Robust standard error	0.042		0.086		0.090		0.029		0.127	
R^2^ / Pseudo R^2^	0.016								0.052	
**Dependent variable: Meat and fish intake**^**1/**^	**Poisson**		**Q (0.25)**		**Q (0.50)**		**Q (0.75)**		**Probit**	
*Number of animals raised for food purposes*										
Coefficient	0.002		0.004	*	−0.001		0.000		0.021	***
Robust standard error	0.002		0.002		0.002		0.003		0.007	
Pseudo R^2^	0.014								0.089	
Number of observations: 313										

Control variables: Age of the household head, youth dependency ratio, average education of the adults of the household, language spoken by the household head (ethnicity), gender of the household head, wealth index, participation in urban jobs, participation in off-farm activities in the community, subsidies and community

*** p value<0.01, ** p value<0.05 and * p value<0.1

^1/^Pseudo R^2^ are not available from quantile regression estimates for count dependent variables such as vegetables intake and meat and fish intake

Source: Author’s survey data (December 2016–April 2017)

Table 5 shows the results from the regressions at the community level. The dimension and significance of the association between home garden diversity and food security measures vary across communities. In the peri-urban community positive associations are only found with plant species diversity, both for food consumption scores (across the whole distribution) and the frequency of vegetables intake. In the semi-rural communities, positive associations are found with both plant species diversity and abundance of animals, particularly in the lowest half of the distribution of food consumption scores. Finally, in the rural community, significant positive associations are only identified with plant diversity in the second quantile of the distribution of food consumption scores and between frequency of meat intake and abundance of animals.

**Table 5:** Association between home garden diversity and food security at the community level, Yucatán, Mexico

	Peri-urban community								Semi-rural communities								Rural community	
**Dependent variable: Food Consumption Score**	**OLS**		**Q(0.25)**		**Q(0.75)**		**Probit**		**OLS**		**Q(0.25)**		**Q(0.50)**		**Probit**		**Q(0.50)**	
*Shannon diversity index (plants)*																		
Coefficient	6.381	**	6.460	**	7.992	**	0.843	**	4.884	*	11.075	***	5.549	*			11.991	**
Standard error	2.757		3.176		3.872		0.428		2.494		3.921		3.195				5.847	
R^2^ / Pseudo R^2^	0.224		0.230		0.130		0.230		0.167		0.150		0.133				0.225	
*Number of animals used for food purposes*																		
Coefficient									0.370	*			0.437	*	0.050	**		
Standard error									0.175				0.237		0.022			
R^2^ / Pseudo R^2^									0.170				0.124		0.229			
**Dependent variable: Vegetables intake**^**1/**^	**Poisson**																	
*Shannon diversity index (plants)*																		
Coefficient	0.156	**																
Robust standard error	0.078																	
R^2^ / Pseudo R^2^	0.021																	
**Dependent variable: Meat and fish intake**^**1/**^																	**Probit**	
*Number of animals raised for food purposes*																	0.067	**
Coefficient																	0.036	
Robust standard error																	0.227	
Pseudo R^2^																		
Number of observations	98		98		98				162		162		162		162		53	

Note: Only significant estimates are reported

Control variables: Age of the household head, youth dependency ratio, average education of the adults of the household, language spoken by the household head (ethnicity), gender of the household head, wealth index, participation in urban jobs, participation in off-farm activities in the community, subsidies and community

*** p value<0.01, ** p value<0.05 and * p value<0.1

^1/^Pseudo R^2^ are not available from quantile regression estimates for count dependent variables such as vegetables intake and meat and fish intake

Source: Author’s survey data (December 2016–April 2017)

From the control variables used in the regression analysis it was found that education, ethnicity, wealth, participation in urban jobs, participation in cash-transfers programmes and urban-rural location are also significant factors in explaining household food security (Table 6). Some of these household characteristics showed an even larger effect than home garden diversity demonstrated. The next subsection examines the interactions between these variables and home garden diversity.

**Table 6:** Determinants of food consumption scores (pooled regressions of the four research sites), Yucatán, Mexico

Dependent variable: Food Consumption Score	OLS		Q (0.25)		Q (0.50)		Q (0.75)		Probit	
	*Coeff.*		*Coeff.*		*Coeff.*		*Coeff.*		*Coeff.*	
*Homegarden characteristics*										
Shannon diversity index	3.894	**	3.250	***	5.865	***	6.104	**	0.276	**
Age of the household head	−0.041		−0.068		−0.036		−0.043		−0.003	
Youth dependency ratio	0.607		1.300		0.682		−0.174		−0.147	
Average education (years)	0.397		0.656		0.878		−0.440	**	0.086	**
*Language spoken by the household head* *(Base category: Spanish and Maya)*										
Maya	3.124		−4.628		12.880	**	−3.065		0.220	
Spanish	3.493		3.191		2.982		6.478	**	−0.106	
Female head (alone)	−3.643				−4.869		−2.343		−0.190	
Male head (alone)	−4.283		−4.197		−4.839		−1.327		−0.333	
Wealth (index)	10.320	**	−6.959	***	6.869		8.375	***	0.678	***
*Rural-urban interactions*			12.077							
Urban jobs	10.528	**	17.402	***	11.003	***	0.391		0.881	***
Off-farm diversification	4.568		5.078		6.483		1.319		0.021	
*Subsidies*										
Sixty-five and over	−0.679		5.038	***	−3.940		−1.759		0.200	
Prospera	1.202		1.481		0.778		0.177		0.210	
*Community (Base category: Hocabá, peri-urban)*										
Sahcabá (semi-rural)	−6.764		−7.966		−4.217		−3.993		−0.650	***
Yaxcabá (semi-rural)	−3.275		−0.859		−3.489		−1.346		−0.409	***
Kancabdzonot (rural)	−4.918		−5.586		−1.686		−4.913		−0.776	***
Constant	60.159	**	45.540	***	53.815	***	78.108	***	0.144	
Pseudo R^2^	0.138		0.119		0.115		0.067		0.162	
Number of observations			313						313	

*** p value<0.01, ** *p* value<0.05 and * p value<0.1

Source: Survey data (December 2016–April 2017)

### Urban jobs

Urban jobs emerged from the analysis as significant factors that explain both food consumption scores and home garden diversity, uncovering trade-offs and complementarities between these two livelihood activities. Households with adults working in urban jobs exhibited a lower diversity of herbs and quantity of animals raised for food purposes but also tended to be better-off in terms of wealth and food consumption scores (Table 7).

**Table 7:** Selected home garden and household characteristics by participation in urban jobs, Yucatán, Mexico

Variable		4 field sites	Hocabá (Peri-urban, sisal region)	Sahcabá (Semi-rural, sisal region)	Yaxcabá (Semi-rural, milpa region)	Kancabdzonot (Rural, milpa region)
Shannon diversity index (all plants, mean)	At least one household member participates in urban jobs	1.796	1.835	1.634	1.912	2.171
	No participation in urban jobs	1.786	1.503	1.372	1.900	2.059
	T-test *p* value	0.891	0.023	0.092	0.937	0.613
Shannon diversity index (herbs used for food purposes, mean)	At least one household member participates in urban jobs	0.395	0.400	0.108	0.699	0.686
	No participation in urban jobs	0.566	0.309	0.185	0.618	0.918
	T-test p value	0.013	0.412	0.389	0.582	0.386
Number of animals raised for food purposes (mean)	At least one household member participates in urban jobs	6.109	5.242	6.152	5.774	13.333
	No participation in urban jobs	8.364	4.375	8.772	7.528	12.068
	T-test p value	0.051	0.689	0.263	0.368	0.783
Wealth index (mean)	At least one household member participates in urban jobs	1.027	1.123	0.945	0.923	0.923
	No participation in urban jobs	0.848	1.005	0.663	0.843	0.843
	T-test p value	<0.001	0.295	0.008	0.445	0.445
Food consumption score (mean)	At least one household member participates in urban jobs	74.420	76.240	71.288	76.596	69.350
	No participation in urban jobs	67.900	72.140	58.430	68.150	74.110
	T-test p value	0.002	0.278	0.007	0.042	0.470

Note: Hocabá, *n* = 92; Sahcabá, *n* = 79; Yaxcabá, *n* = 78; Kancabdzonot, *n* = 53

Source: Author’s survey data (December 2016–April 2017)

The decision to work in urban areas has important implications for the mean wage that individuals can aspire to earn. From the survey data collected, individuals working inside their communities reported an average monthly wage of MXN 990.6 (GBP 40.4; USD 52.7), while those working outside their communities reported an average wage more than four times greater (Table 8). Although urban jobs provide opportunities to earn better incomes, access to these jobs is constrained due to context-specific formal and informal institutions (Ellis 1998; Becker 2013; Tacoli 1998).

**Table 8:** Characteristics of working adults by location of occupation, Yucatán, Mexico

Characteristics	Outside the community	Inside the community	T-test/Chi-squared *p* value
Mean age	34.38	47.14	<0.001
Mean years of education	8.71	6.02	<0.001
Proportion of women	14.84	85.16	<0.001
Proportion of men	44.74	55.26	<0.001
Proportion of Maya speakers	6.38	93.62	<0.001
Proportion of Maya and Spanish speakers	27.9	72.1	<0.001
Proportion of Spanish speakers	41.14	58.86	<0.001
Mean monthly wage, MXN	4358.23	990.63	<0.001
(GBP/USD)	(177.73/232.07)	(40.40/52.75)	

Observations: Hocabá 337, Sahcabá 270, Yaxcabá 225, Kancabdzonot 144

Source: Author’s survey data (December 2016–April 2017)

In the research sites, young adults, men, more educated people and Spanish speakers are more likely to participate in urban jobs. In contrast, occupations within the communities are more accessible livelihood strategies for elderly people, women and Mayan speakers. These differences are likely due to the higher education and Spanish-speaking requirements of urban jobs in comparison with on-farm and off-farm occupations inside the communities. Furthermore, because of the patriarchal nature of Mexican society, occupations are more socially acceptable for women if they are located within their own communities than if they are based in urban area; local occupations allow women to spend more time in their communities and take care of their multiple house chores.

## Discussion

The analysis above provides evidence on the contribution of home garden diversity to food security and sheds light on how the rural–urban location of the household and the participation in urban jobs mediate this relationship. Differences in the size of the coefficients across quantile regressions are likely reflecting how home garden plant diversity plays a complementary rather than a main role in household food consumption. Larger effects were identified at the highest quantiles of the FCS distribution which grouped households that also tended to be wealthier. In contrast, the abundance of animals was more relevant in the lowest quantiles of the distribution of both FCS and the frequency of meat intake. These findings are relevant for the design and targeting of home garden interventions; while most of the households may benefit from increased access to inputs and training to grow vegetables, increased access to poultry and small livestock components are likely to result in greater impacts among the poorest households.

Household rural–urban interactions can lead to the introduction of new species and gardening techniques as well as to the loss of biodiversity and traditional knowledge (De Haan 1999; Guerrero Peñuelas 2007; Cano-Ramirez et al. 2012). This research identified that closer interactions with urban areas tend to increase the adoption of ornamental plants but also favour overall plant diversity levels in the most urbanised communities. Wiersum (2006) arrives at similar conclusions on the relationship between ornamental plants and off-farm jobs in a review of studies on Indonesian home gardens; he finds that, when alternative income opportunities emerged, households tended to increase the production of ornamental plants in their home gardens. However, this pattern is likely to reduce the resilience of the household in case of job loss as well as undermine the role of the home garden as a biocultural repository.

Participation in urban jobs was identified as having positive associations with the overall levels of plant diversity in the most urbanised communities, but negative associations with the diversity of vegetables and other herbs used for food purposes and with the abundance of animals. Positive associations with plant diversity are likely explained by the introduction of new species and availability of resources to invest in the home garden. Synergy between on-farm and off-farm occupations has been identified in previous studies. For example, in Ghana, Tsiboe et al. (2016) find positive effects of off-farm work on nutrient availability, noting that households that combined off-farm business with farming showed the greatest food nutrient availability. This is particularly relevant for semi-rural communities, where households tend to depend on both on-farm and off-farm livelihoods.

Negative associations between urban jobs and both herb diversity and animal abundance in home gardens may be indicative of trade-offs in the time allocated between on-farm and off-farm activities. The processes of growing herbs and raising animals tend to be more time-consuming in comparison with taking care of other home garden components, such as fruit trees. Guerra Mukul (2005) arrives at a similar conclusions from a study conducted in Yaxcabá, Mexico: the engagement of the household head in urban paid jobs caused a reduction in *milpa* production; the loss of knowledge transmission between fathers and children; and the deterioration of the home garden facilities, such as fences, pigsties and poultry pens. Nonetheless, complementarities are also identified where urban jobs may be allowing households to invest in their home gardens; this appears to apply to total plant diversity, particularly in the communities located in the sisal region.

Participation in urban jobs, however, is not an option for all households nor for all household members as explained in section 3. Previous studies similarly have found that access to non-agricultural occupations is constrained by context-specific formal and informal institutions, which are based on political power, political affiliation, religion, income, ethnicity, gender, generation, and other sociodemographic characteristics (Ellis 1998; Tacoli 1998; Becker 2013). It has been observed in different developing countries across Asia (Hoang et al. 2008; Rungmanee 2014), Africa (Adusah-Karikari 2015) and Latin America (Bravo-Ureta et al. 1996; Guerrero Peñuelas 2007), that young and better-educated family members are more likely to migrate and to take advantage of non-farm job opportunities.

## Conclusions

This research contributes to the understanding of how household characteristics and agrobiodiversity mediate the impact of home gardens on food security. Positive associations are found between plant diversity and animal abundance and different measures of food security. The dimension and the significance of these positive associations was found to vary depending on the type of home garden diversity, across communities and across quantiles of the distribution of food security measures. Plant diversity was significant across all the peri-urban–rural spectrum, while animal abundance was significant in the semi-rural and rural communities, particularly in the lowest quantile of the distribution of food consumption scores.

In the research sites, home gardening interacts with other livelihood activities, such as urban jobs, in the fulfilment of household food consumption needs. Participation in urban jobs, however, is constrained by formal and informal institutions. In the households studied, younger individuals, better–educated people, males and Spanish speakers are more likely to engage in jobs in urban areas. Engagement in urban jobs was found to involve not only complementarities with the overall plant diversity of home gardens, but also trade-offs with the diversity of vegetables and other herbs used for food purposes and with the abundance of animals raised for food purposes.

One of the main limitations of this study is its reliance on self-reporting measures of food security which are subject to recall errors. Nonetheless, the quality of the data collected was ensured through following international standards, such as those that the World Food Programme has established for the collection and analysis of the data to construct food consumption scores. In consideration of the high incidence of malnutrition in the region, both in form of undernutrition and overweight and obesity, future research could explore the role of home gardening interventions on nutrition status and the incidence of chronic diseases. The relevance of studying this topic has become apparent during the COVID-19 pandemic, which has disproportionately affected populations who suffer from obesity and chronic diseases such as diabetes and hypertension (Hernandez, 2020). Other areas for future research include the study of the contribution of home gardening to food security across different seasons and intra-household dynamics of food consumption, as well as the provision of non-tangible services, such as those social and cultural.

## Supplementary information

ESM 1 (PDF 532 kb)

## Footnotes

1 Wald test of exogeneity (correlation = 0), *p* value>0.400 for all the different specifications.

## References

[CR1] Adjimoti GO, Kwadzo GTM (2018). Crop diversification and household food security status: Evidence from rural Benin. Agriculture & Food Security.

[CR2] Adusah-Karikari A (2015). Black gold in Ghana: Changing livelihoods for women in communities affected by oil production. Extractive Industries and Society-an International Journal.

[CR3] Baliki G, Brück T, Schreinemachers P, Uddin MN (2019). Long-term behavioural impact of an integrated home garden intervention: Evidence from Bangladesh. Food Security.

[CR4] Becerril J, Castañeda J, Solís C (2014). Pobreza, agrodiversidad y nutrición en el Yucatán rural, 2010. Avances en Investigación Agropecuaria.

[CR5] Becker LC (2013). Land sales and the transformation of social relations and landscape in peri-urban Mali. Geoforum.

[CR6] Bellon M, Ntandou-Bouzitou G, Caracciolo F (2016). On-farm diversity and market participation are positively associated with dietary diversity of rural mothers in southern Benin, West Africa. PLoS One.

[CR7] Bhagowalia P, Headey D, Kadiyal S (2012). Agriculture, income, and nutrition linkages in India: Insights from a nationally representative survey. *Discussion Paper*.

[CR8] Bravo-Ureta BE, Quiroga RE, Brea JA (1996). Migration decisions, agrarian structure, and gender: The case of Ecuador. Journal of Developing Areas.

[CR9] Cabalda AB, Rayco-Solon P, Solon JAA, Solon FS (2011). Home gardening is associated with Filipino preschool children's dietary diversity. Journal of the American Dietetic Association.

[CR10] Cano-Ramirez M, De la Tejera B, Casas A, Salazar L, Garcia-Barrios R (2012). Human migration and home gardens in an indigenous community in Central Mexico. Botanical Sciences.

[CR11] Chávez Zander, U. (2014). Agrobiodiversity, cultural factors and their impact on food and nutrition security: A case-study in the south-east region of the Peruvian Andes.

[CR12] Chi Quej J d l Á (2009). Caracterización y manejo de los huertos caseros familiares en tres grupos étnicos (Mayas peninsulares, Choles y Mestizos) del Estado de Campeche, México.

[CR13] Consejo Nacional de Evaluación de la Política de Desarrollo Social. (2012). Pobreza en México. Resultados de pobreza en México 2012 a nivel nacional y por entidades federativas. http://coneval.gob.mx/. Accessed 03 Feb 2014.

[CR14] Consejo Nacional de Evaluación de la Política de Desarrollo Social. (2014). La pobreza en la población indígena de México, 2012. México, Distrito Federal.

[CR15] Cuanalo de la Cerda H, Llanes Chan W, Hernández Montero I, Canul Ku J, Ek Dzul B, Uicab Couoh A, Pedroza Sandoval A, Ruiz Torres J, Alaniz Gutiérrez L (1998). El desarrollo rural perdurable en Yucatán. *Desarrollo rural sustentable. Experiencias, enfoques y perspectivas* (pp. 78–88).

[CR16] De Haan A (1999). Livelihoods and poverty: The role of migration - A critical review of the migration literature. Journal of Development Studies.

[CR17] Dewey K (1981). Nutritional consequences of the transformation from subsistence to commercial agriculture in Tabasco, Mexico. Human Ecology.

[CR18] Dietrich J (2011). Gendered division of labour in homegardens in Calakmul, Campeche, Mexico.

[CR19] Ellis F (1998). Household strategies and rural livelihood diversification. Journal of Development Studies.

[CR20] FAO, I., UNICEF, WFP and WHO (2019). The State of Food Security and Nutrition in the World 2019. Safeguarding against economic slowdowns and downturns.

[CR21] Fernandes ECM, Nair PKR (1986). An evaluation of the structure and function of tropical homegardens. Agricultural Systems.

[CR22] Food and Agriculture Organization, FAO (2008). An introduction to the basic concepts of food security. *Food Security Information for Action. Practical Guides*.

[CR23] Guerra Mukul R (2005). *Factores sociales y económicos que definen el sistema de producción de traspatio en una comunidad rural de Yucatán, México*.

[CR24] Guerrero Peñuelas AG (2007). The impact of migration on housegardens management in La Purísima Concepción Mayorazgo, San Felipe del Progreso, state of Mexico. Investigaciones Geográficas.

[CR25] Hirvonen K, Hoddinott J (2017). Agricultural production and children's diets: Evidence from rural Ethiopia. Agricultural Economics.

[CR26] Hoang XT, Dinh TP, Nguyen TH (2008). Urbanisation and rural development in Vietnam's Mekong Delta: Livelihood transformations in three fruit-growing settlements. *Working Paper Series on Rural-Urban Interactions and Livelihood Strategies*.

[CR27] Instituto Nacional de Estadística y Geografía. (2010). *Censo de Población y Vivienda 2010*. INEGI. http://inegi.org.mx/. Accessed 08 Feb 2014.

[CR28] Instituto Nacional de Salud Pública (2013). *Encuesta Nacional de Salud y Nutrición 2012*. http://ensanut.insp.mx/. Accessed 29 Jan 2014.

[CR29] Jiménez-Osornio JJ, Morales R, M. d. R., & Aké Gómez, A., Jarvis DI, Sevilla-Panizo R, Chavez-Servia JL, Hodgkin T (2003). Mayan home gardens: Sites for *in situ* conservation of agricultural diversity. Seed systems and crop genetic diversity on-farm., Pucallpa, Peru., 2003.

[CR30] Jones AD, Shrinivas A, Bezner-Kerr R (2014). Farm production diversity is associated with greater household dietary diversity in Malawi: Findings from nationally representative data. Food Policy.

[CR31] Kay C (2015). The agrarian question and the neoliberal rural transformation in Latin America. European Review of Latin American and Caribbean Studies.

[CR32] Kontoleon A, Pascual U, Smale M, Kontoleon A, Pascual U, Smale M (2009). Introduction: Agrobiodiversity for economic development: What do we know?. Agrobiodiversity conservation and economic development.

[CR33] Kumar BM, Nair PKR (2004). The enigma of tropical homegardens. Agroforestry Systems.

[CR34] Kumar N, Harris J, Rawat R (2015). If they grow it, will they eat and grow? Evidence from Zambia on agricultural diversity and child undernutrition. The Journal of Development Studies.

[CR35] Leatherman TL, Goodman A (2005). Coca-colonization of diets in the Yucatan. Social Science & Medicine.

[CR36] Lovon M, Mathiassen A (2014). Are the world food Programme’s food consumption groups a good proxy for energy deficiency?. Food Security.

[CR37] Luna-González D, Sørensen M (2018). Higher agrobiodiversity is associated with improved dietary diversity, but not child anthropometric status, of Mayan Achí people of Guatemala. Public Health Nutrition.

[CR38] Malapit HJL, Kadiyala S, Quisumbing AR, Cunningham K, Tyagi P (2015). Women's empowerment mitigates the negative effects of low production diversity on maternal and child nutrition in Nepal. The Journal of Development Studies.

[CR39] Mariaca Méndez R, Mariaca Méndez R (2012). La complejidad del huerto familiar maya del sureste de México. El huerto familiar del sureste de México.

[CR40] Marsh R (1998). Building on traditional gardening to improve household food security. Food, Nutrition and Agriculture.

[CR41] Masset E, Haddad L, Cornelius A, Isaza-Castro J (2012). Effectiveness of agricultural interventions that aim to improve nutritional status of children: Systematic review. British Medical Journal.

[CR42] Moreno-Calles, A. I., Toledo, V., & Casas, A. (2014). Los sistemas agroforestales tradicionales de México: Una aproximación biocultural. *Botanical Sciences, 91*(4), 375. 10.17129/botsci.419.

[CR43] Moreno-Calles AI, Casas A, Rivero-Romero AD, Romero-Bautista YA, Rangel-Landa S, Fisher-Ortiz RA (2016). Ethnoagroforestry: Integration of biocultural diversity for food sovereignty in Mexico. Journal of Ethnobiology and Ethnomedicine.

[CR44] Musotsi AA, Sigot AJ, Onyango MOA (2008). The role of home gardening in household food security in Butere division of western Kenya. African Journal of Food, Agriculture, Nutrition and Development.

[CR45] Olney DK, Bliznashka L, Pedehombga A, Dillon A, Ruel MT, Heckert J (2016). A 2-year integrated agriculture and nutrition program targeted to mothers of young children in Burkina Faso reduces underweight among mothers and increases their empowerment: A cluster-randomized controlled trial. The Journal of Nutrition.

[CR46] Pietersen S, López-Acosta JC, Gomez-Díaz JA, Lascurain-Rangel M (2018). Floristic diversity and cultural importance in agroforestry systems on small-scale farmer’s livelihoods in Central Veracruz. México. Sustainability.

[CR47] Pingali P (2007). Westernization of Asian diets and the transformation of food systems: Implications for research and policy. Food Policy.

[CR48] Ruel MT, Quisumbing AR, Balagamwala M (2018). Nutrition-sensitive agriculture: What have we learned so far?. Global Food Security.

[CR49] Rungmanee S (2014). The dynamic pathways of agrarian transformation in the northeastern Thai-Lao borderlands. Australian Geographer.

[CR50] Schreinemachers P, Patalagsa M, Islam M, Uddin M, Ahmad S, Biswas S (2015). The effect of women’s home gardens on vegetable production and consumption in Bangladesh. Food Security.

[CR51] Sibhatu KT, Qaim M (2018). Review: Meta-analysis of the association between production diversity, diets, and nutrition in smallholder farm households. Food Policy.

[CR52] Sibhatu KT, Krishna VV, Qaim M (2015). Production diversity and dietary diversity in smallholder farm households. Proceedings of the National Academy of Sciences.

[CR53] Soemarwoto O, Steppler HA, Nair PKR (1987). Homegardens: A traditional agroforestry system with a promising future. Agroforestry. A decade of development.

[CR54] Soemarwoto O, Soemarwoto I, Karyono S, E. M., & Ramlan, A. (1985). The Javanese home garden as an integrated agro-ecosystem. Food and Nutrition Bulletin.

[CR55] Stuart JW (1993). Contribution of dooryard gardens to contemporary Yucatecan Maya subsistence. Biotica.

[CR56] Sunwar S, Thornström C-G, Subedi A, Bystrom M (2006). Home gardens in western Nepal: opportunities and challenges for on-farm management of agrodiversity. Biodiversity and Conversation.

[CR57] Tacoli C (1998). Bridging the divide: Rural-urban interactions and livelihood strategies.

[CR58] Terán S, Rasmussen CH (1994). La milpa de los mayas: la agricultura de los mayas prehispánicos y actuales en el noreste de Yucatán.

[CR59] Tesfamariam BY, Owusu-Sekyere E, Emmanuel D, Elizabeth TB (2018). The impact of the homestead food garden programme on food security in South Africa. Food Security.

[CR60] Tsiboe F, Zereyesus YA, Osei E (2016). Non-farm work, food poverty, and nutrient availability in northern Ghana. Journal of Rural Studies.

[CR61] Wiersum KF, Kumar BM, Nair PKR (2006). Diversity and change in home garden cultivation in Indonesia. *Tropical homegardens. A time-tested example of sustainable agroforestry* (Vol. 3, pp. 13-24, advances in agroforestry).

[CR62] Wiesmann D, Bassett L, Benson T, Hoddinott J (2009). Validating the world food Programme’s food consumption score and alternative indicators of household food security. *Discussion Paper* 00870.

[CR63] World Bank (2007). From agriculture to nutrition: Pathways, synergies and outcomes.

[CR64] World Food Programme (2008). Food consumption analysis. Calculation and use of the food consumption score in food security analysis.

[CR65] Zanello G, Shankar B, Poole N (2019). Buy or make? Agricultural production diversity, markets and dietary diversity in Afghanistan. Food Policy.

