# Supplementary material for: Homegarden diversity and food security in southern Mexico
Source: Food Secur. 2021 Feb 13;13(3):669–83. doi: 10.1007/s12571-021-01148-w (PMC7881320; doi:10.1007/s12571-021-01148-w)
Supplement: Supplementary file 1 — (PDF 532 kb) [file 12571_2021_1148_MOESM1_ESM.pdf]

Household survey questionnaire

Introduction and asking for consent
1. Household ID
2. Date
3. Name of the interviewer
4. Municipality
5. Community
4. Household address
5.Is the house inhabited?

Yes ☐ No ☐

6.Respondant's name
7.Was this household surveyed in 1997?

Yes ☐ No ☐

If it was not Who is now living in the house? ____________________

I. HOMEGARDEN CHARACTERISTICS

8. Solar size (square meters)
9. Homegarden age

☐ 15 or less
☐ Between 16 and 50
☐ Between 51 and 100
☐ More than 100

10. What is(are) the source(s) of the water used for watering the homegarden?
Code: 1=well, 2=tap water, 3=rain water, 4=none.
11. What vegetal species do you grow in your homegarden?

Species name	Number	Main uses	Gift or exchange (Yes / No)	Sale (Yes / No)	Where do you sale it?	If other community, specify the name	Price (Mexican pesos)	Unit of measurement	How often do you sale it?

Codes
Main uses: 1=eating fresh, 2=condiment, 3=cooking, 4=drinking, 5=ornamental, 6=medicinal, 7=construction, 8=animal feeding, 9=fodder, 10=fertilizer, 11=shadow, 12=tool / utensil, 13=wood, 14=insecticide, 15=ritual, 16=to attract bees / birds, 17=other.
Sale: 0= Not for sale, 1= Sold fresh, 2=Sold cooked.
Where: Butcher shop, market, mill, groceries shop, house, street_sale, other community.
Frequency: Weekly, monthly, yearly, occasionally, seasonal, other

12. Is there any fruit or crop loss?

Yes ☐ No ☐

If yes

12a. Which species? ____________________

12b. Why is it wasted? (no_time, no_help, do_not_like, lowprices, plague, other) ____________________

13. Do you grow crops or fruit trees in another plot (different from the homegarden and the milpa)?

Yes [ ] No [ ]

If yes

13a. Main use

Consumption [ ] Sale [ ]

14. What animals do you rise in your homegarden?

Species name	Number	Main uses	Gift or exchange (Yes / No)	Sale (Yes / No)	Where do you sale it?	If other community, specify the name	Price (Mexican pesos)	Unit of measurement	How often do you sale it?

Codes

Main uses: 1=food, 2=ritual, 3=sale, 4=fertilizer, 5=pet, 6=hunting, 7=protection.

Sale: 0= Not for sale, 1= Sold alive, 2=Sold processed.

Frequency: Weekly, monthly, yearly, occasionally, seasonal, other

Where: Butcher shop, market, mill, groceries shop, house, street_sale, other community.

15. Do you use any of these inputs in your homegarden?

Input	Yes/No	Source	Where	Community name	Amount spent	How often do you buy it?
Seeds						
Earth						
Fertilizer						
Herbicide						
Pesticide						
Animal feeding						
Animal medicines / veterinary services						

Codes:

Source: 1=do it by a family member, 2=gift/exchange, 3=government, 4=university, 5=NGO, 5=bought.

Where: 1=inside the comunity, 2=outside the community

Frequency: Weekly, monthly, yearly, occasionally, seasonal, other

16. What are the main differences between this homegarden and the homegarden you had as a child?

II. FOOD SECURITY

17. I would like to ask you about all the different foods that your household members have eaten in the last 7 days. Could you please tell me how many days in the past week your household has eaten the following foods?
(for each food, ask what the primary source of each food item eaten that week was, as well as the second main source of food, if any)

	Number of days (0-7)	Primary source	Secondary source
**Maize**: Maize, pozol, tortilla, pimitos, tamales or any other meal made of maize			
**Tubers**: Tannia, potato, cassava, kohlrabi, etc.			
**Bread / wheat**: Rice, bread, pasta, oat or breakfast cereal.			
**Pulses and groundnuts**: Beans, lentils, peanuts, wax beans, slender green beans, broad beans or nuts.			
**Milk and dairy products**: Milk, cheese, cream or yogurth			
**Meat**: Pork, beef, chicken, etc. (Do not include organs of these animals)			
**Organ meat:** Liver, kidney, heart or other organs.			
**Fish and seafood**			
**Eggs**			
**Orange vegetables:** Squash, carrot, sweet potato or red pepper			
**Dark green vegetables**: Chaya, spinach, amaranth, chard or broccoli.			
**Other vegetables**: cucumber, tomato, cabbage, etc. (Do not include condiments)			
**Orange fruits**: Mango, papaya, melon.			
**Other fruits**: Citrus, banana, plum, watermelon, pitahaya, anonas, cocoyol, etc.			
**Fat:** Oil, animal fat or butter			
**Sugar:** Sweets, cakes, biscuits, mermelade or soft drinks			
**Condiments:** Small amounts of chilli, onion, coriander, tomato, sugar, coffee			

Codes
Sources: 1=hunting, 2=buy, 3=milpa, 4=forest, 5=gift, borrowing or exchange, 6=homegarden, 7=other plot, 8=other (specify)

18. Have you or any of the household members skipped a meal because of the lack of food or money? (In the last three months)

Yes ☐ No ☐

19a. If yes, who? ______________________

19b. If yes, Why did you face lack of food or money? ______________________

III. HOUSEHOLD CHARACTERISTICS

20. What type of toilet facilities are available? (1= flush toilet, 2=letrine, 3=outdoors)

21. Which of these services, appliances or equipment do you have?

	No=0, Yes=1
Tap water	
Electricity	
Refrigerator	
TV	
Radio	
Mobile	
Fan	
Bicycle	
Motorcycle	
Car	

22. How many families live in the household? ______________________

23. How many adults live in the household? ______________________

25. How many children and young people below 18 years old? __________

26. Household members characteristics

Member ID (1 head, 2 spouse)	First name	First surname	Second surname	Relationship to household head	Sex	Age	Marital status	Language	Education
1									
2									
3									
4									
5									
6									
7									

Codes:
Relationship: 1=spouse, 2=son/daughter, 3=parents, 4=grandparents, 5=grandchild, 6=daughter/son in law, 7=other relative, 8=non relative.
Sex= 0=male, 1=female.
Marital status: 1=single, 2=married, 3=cohabitating, 4=divorced, 5=widow/er.
Language: 0=Spanish, 1=Maya, 3=Maya and Spanish, 4=Other.
Education: Number of years, 0 = illiterate people.

27. Household members health status

Member ID (1 head, 2 spouse)	Chronic diseases	Infectious diseases	Other health conditions	Access to health services
1				
2				
3				
4				
5				
6				
7				

Codes:
Chronic disease: 0=none, 1=heart disease, 2=diabetes, 3=asthma, 4= hypertension, 5=arthritis, 6=stroke, 7=cancer, 8=other.
Infectious disease: 0=none, 1=respiratory, 2=gastro-intestinal, 3=dengue, 4=zika, 5=chikungunya, 6=other.
Access to health services: 0=none, 1=seguro popular, 2=servicios de salud (SSA), 3=IMSS, 4=ISSSTE, 5=private, 6=other.

IV. LIVELIHOODS AND RURAL-URBAN LINKS

28. What are the occupations of the household members?

Member ID (1 head, 2 spouse)	Main occupation	Other occupations	Where do you perform these activities?	If outside the community. Where?	How often do you commute back to the community?	How much do you spend in transportation?	How often do you spend this amount?	Do you receive any income from this occupation? (No=0, Yes=1)	How often do you receive this income?	How much do you receive?
1										
2										
3										
4										
5										
6										
7										

Codes
Occupations: 1=agriculture (milpa or plot), 2=apiculture, 3=livestock, 4=homegarden, 5=hunting, 6=wood cutting, 7=fisher, 8=housewife, 9= student, 10=clothes making, 11=other handicraft, 12=taxi (bike or motorcycle), 13=taxi (car), 14=merchant, 15=trade, 16=housekeeper, 17=construction worker, 18=labourer, 19=professional, 20=government employee,
Where: 0=within the community, 1=outside the community.
Frequency= 1=everyday, 2=twice or three times a week, 3=weekely, 4=every 15 days, 5=monthly, 6=once the work is finished, 7=other(specify).

28a. If the milpa was cultivated in the household What was the area cultivated in 2016?
28b. What crops did you cultivate?

29. Did you receive any support (subsidy or funding) from the government / university or NGO in the last year?

Type of support	Source	Source name (Programme and/or	Amount or support details	How often do you receive this support?

Codes
Type: 1=in kind, 2=money, 3=technical assistance, 4=products commercialization, 5=other(specify).
Source: 1=government, 2=university, 3=NGO, 4=other.
How often= 1=weekely, 2=every 15 days, 3=monthly, 4=every two months, 5=every six months, 6=yearly.

30. Have any of the household members receive or send remittances?

Member ID (1 head, 2 spouse)	Receive (0=No, 1=Yes)	How much?	How often?	From who?	Where does this person live?
1					
2					
3					
4					
5					
6					
7					

Codes
How often= 1=weekely, 2=every 15 days, 3=monthly, 4=every two months, 5=every six months, 6=yearly.
Who: 1=family, 2=friends, 3=other (specify)

Member ID (1 head, 2 spouse)	Send (0=No, 1=Yes)	How much?	How often?	Whom?	Where does this person live?
1					
2					
3					
4					
5					
6					
7					

Codes
How often= 1=weekely, 2=every 15 days, 3=monthly, 4=every two months, 5=every six months, 6=yearly.
Whom: 1=family, 2=friends, 3=other (specify).

31. Is there any family member who used to live in the house but migrated to another community?

Yes ☐ No ☐

31a. How many former household members? ____________

31b.Where do they live? ____

V. PERCEPTIONS AND OPINIONS

32. What are the main changes the community has faced in the last 10 years?

____

____

33. What do you think is to have a 'good life' in the community?

	Attending to school
	Good social relationships
	Food sovereignty
	Food quality
	Having no worries
	Health
	Food (access)
	Job
	Shelter / House
	Other ____________

34. Would you like to have more plants or animals in you homegarden?

Yes ☐ No ☐

Which species? ____

Why? ____

Why? ____

____

Thank you very much for your time. May I take a picture of your homegarden?
I brought you these seeds (radish and coriander) as a way to thank for your time and knowledge.

General comments.
